# Determinants of door-in-door-out time in patients with ischaemic stroke transferred for endovascular thrombectomy

**DOI:** 10.1177/23969873231177768

**Published:** 2023-05-30

**Authors:** Ruben M van de Wijdeven, Martijne HC Duvekot, Patrick J van der Geest, Walid Moudrous, Kirsten RIS Dorresteijn, Annemarie D Wijnhoud, Laus JMM Mulder, Kees CL Alblas, Nabil Asahaad, Henk Kerkhoff, Diederik WJ Dippel, Bob Roozenbeek

**Affiliations:** 1 Department of Neurology, Erasmus MC University Medical Center, Rotterdam, the Netherlands; 2 Department of Neurology, Albert Schweitzer Hospital, Dordrecht, the Netherlands; 3 Ambulance Rotterdam-Rijnmond, Barendrecht, the Netherlands; 4 Department of Neurology, Maasstad Hospital, Rotterdam, the Netherlands; 5 Department of Neurology, Franciscus Gasthuis & Vlietland, Rotterdam, the Netherlands; 6 Department of Neurology, IJsselland Hospital, Capelle aan den IJssel, the Netherlands; 7 Department of Neurology, Ikazia Hospital, Rotterdam, the Netherlands; 8 Department of Neurology, Van Weel-Bethesda Hospital, Dirksland, the Netherlands

**Keywords:** Ischaemic stroke, door-in-door-out time, endovascular thrombectomy, hospital workflow

## Abstract

**Background:**

Long door-in-door-out (DIDO) times are an important cause of treatment delay in patients transferred for endovascular thrombectomy (EVT) from primary stroke centres (PSC) to an intervention centre. Insight in causes of prolonged DIDO times may facilitate process improvement interventions. We aimed to quantify different components of DIDO time and to identify determinants of DIDO time.

**Methods:**

We performed a retrospective cohort study in a Dutch ambulance region consisting of six PSCs and one intervention centre. We included consecutive adult patients with anterior circulation large vessel occlusion, transferred from a PSC for EVT between October 1, 2019 and November 31, 2020. We subdivided DIDO into several time components and quantified contribution of these components to DIDO time. We used univariable and multivariable linear regression models to explore associations between potential determinants and DIDO time.

**Results:**

We included 133 patients. Median (IQR) DIDO time was 66 (52–83) min. The longest component was CTA-to-ambulance notification time with a median (IQR) of 24 (16–37) min. DIDO time increased with age (6 min per 10 years, 95% CI: 2–9), onset-to-door time outside 6 h (20 min, 95% CI: 5–35), M2-segment occlusion (15 min, 95% CI: 4–26) and right-sided ischaemia (12 min, 95% CI: 2–21).

**Conclusions:**

The CTA-to-ambulance notification time is the largest contributor to DIDO time. Higher age, onset-to-door time longer than 6 h, M2-segment occlusion and right-sided occlusions are independently associated with a longer DIDO time. Future interventions that aim to decrease DIDO time should take these findings into account.

## Introduction

Over the last decades, vast efforts have been made to improve care for acute ischaemic stroke patients. The benefit of reperfusion therapies such as intravenous thrombolytics (IVT) and endovascular thrombectomy (EVT) has been well established, but their effects diminish with increasing time since onset.^1–5^ Treatment with IVT is readily available in most hospitals, but it is generally less effective than EVT in patients with ischaemic stroke caused by large vessel occlusion (LVO). EVT, on the other hand, is effective in patients with LVO, but it is only available in specialized intervention centres.^6^ In current practice, suspected stroke patients are usually transported to the nearest hospital for rapid administration of IVT. When patients turn out to be EVT-eligible, they must then be transferred to an intervention centre for EVT (‘drip-and-ship’ strategy). These interhospital transfers are an important cause of treatment delay in EVT.^7,8^

Another important contributor to this treatment delay in EVT is the door-in-door-out (DIDO) time, a time metric encompassing the duration of patient stay at the PSC. DIDO time is relatively overlooked compared to other workflow metrics in acute stroke, despite its twofold importance. First, decreasing DIDO time leads to shorter onset-to-groin times in LVO patients primarily presented at a PSC, which may lead to better outcomes.^9^ Second, DIDO time may play a role in making informed decisions on prehospital transportation strategies for suspected stroke patients. Shorter DIDO times make transportation of a suspected stroke patient to a PSC for faster initiation of diagnostic workup and treatment with IVT more viable.^10^ This balance may be further shifted towards favouring transportation to a PSC with the heralding of new thrombolytic agents which may yield higher recanalization rates in patients with LVO.^11^ In this study, we aimed to quantify the components of DIDO time and to identify determinants of DIDO time, in order to facilitate process improvement interventions in the future.

## Methods

### Study design

We performed a retrospective observational cohort study within one Dutch ambulance region consisting of one intervention centre and six referring PSCs with a catchment area of ±1,300,000 inhabitants. Working arrangements within this region dictated drip-and-ship transportation strategy for suspected stroke patients and there were no prehospital stroke scales implemented over the inclusion period. This study was conducted in accordance with the Dutch Agreement on Medical Treatment Act and the European General Data Protection Regulation. The Institutional Review Board of the Erasmus MC University Medical Center has reviewed the study protocol and confirmed that the Dutch Medical Research Involving Human Subjects Act is not applicable (MEC-2022-0013). This study is conducted and reported according to the STROBE guideline. The checklist can be found in the Supplemental Appendix.

### Study population and procedures

We identified all consecutive patients with acute ischaemic stroke who were presented by ambulance at the emergency department (ED) of the intervention centre between October 1, 2019, and November 30, 2020. Inclusion criteria for our study were ⩾18 years of age, diagnosis of acute ischaemic stroke caused by anterior circulation LVO, primary presentation at an ED of a PSC followed by transfer to the intervention centre for EVT and availability of door-in and door-out times from ambulance logs.

Door-in time was defined as the opening of ambulance door at the ED (automatic logging). Door-out time was defined as the moment of ambulance departure for interhospital transfer (manual logging). Door-in, ambulance notification and door-out times were collected from the ambulance call reports. Imaging acquisition times of non-contrast-enhanced computed tomography scan (NCCT), computed tomography angiography (CTA) and computed tomography perfusion (CTP) were acquired from imaging source data. Imaging acquisition times and ambulance notification times were used to divide DIDO time into components: door-to-NCCT time, NCCT-to-CTA time, CTA-to-ambulance notification time and notification-to-departure time. We specified potential determinants based on current literature: sex, age, pre-stroke modified Rankin Scale (mRS), time and type of onset, baseline National Institutes of Health Stroke Scale (NIHSS) score, systolic blood pressure (SBP), diastolic blood pressure (DBP), referring PSC, acquisition of CTP, administration of IVT and location of occlusion. Data on these potential determinants were collected from the hospital electronic medical record systems or the ambulance call reports. In addition to these clinical characteristics, we included the following hospital-specific logistical and procedural characteristics: location of initial patient workup (CT-room or ED-room), location of CT-room (ED or elsewhere in hospital), transfer notification order (intervention centre first or ambulance service first) and stroke alarm notification by ambulance (notification of neurologist or notification of ED). Onset-to-door time was dichotomized on a 6h cut-point, as this marks the end of the conventional treatment window for EVT in acute ischaemic stroke. Hypertension was defined as SBP > 185 mmHg or DBP > 110 mmHg, as these values are the upper limits for safe administration of IVT to patients with ischaemic stroke as defined by national guidelines.^12^ Office hours were defined as periods between 8 a.m. and 5 p.m. on weekdays, excluding national holidays. Pre-stroke mRS was categorized into independent (mRS 0–2) and dependent (mRS 3–5) functional performance. NIHSS at the intervention centre was used as a measure of stroke severity. As there were only few internal carotid artery terminus (ICA-T) occlusions, they were grouped with M1-occlusions for the analysis.

### Sample size calculation

The number of regression coefficients in the multivariable linear mixed regression model is determined by the sample size. To allow an estimated 10 covariates to be included into the multivariable regression model, we needed to include at least 100 patients.

### Statistical analysis

Categorical variables were reported as numbers and percentages. Normally distributed continuous data were reported as means with standard deviations (±SD). Non-normally distributed continuous data were reported as median values with interquartile range (IQR). DIDO time was subdivided into several components in patients with available imaging acquisition times. We used means instead of medians for this analysis, to ensure all components added up to form the mean DIDO time. For the descriptive analyses we restricted the analyses to complete cases.

The association between potential determinants and DIDO time was analysed using univariable linear regression analyses. Finally, we performed linear mixed model regression analyses using age, sex and variables with a *p*-value <0.20 in univariable linear regression as fixed effects and PSC as random effect. Missing values were replaced with multiple imputation (*n* = 5 imputation sets) for the regression analyses. All analyses were performed in R (version 4.2.1) and Rstudio (version 2022.07.1).

## Results

**Figure 1.**
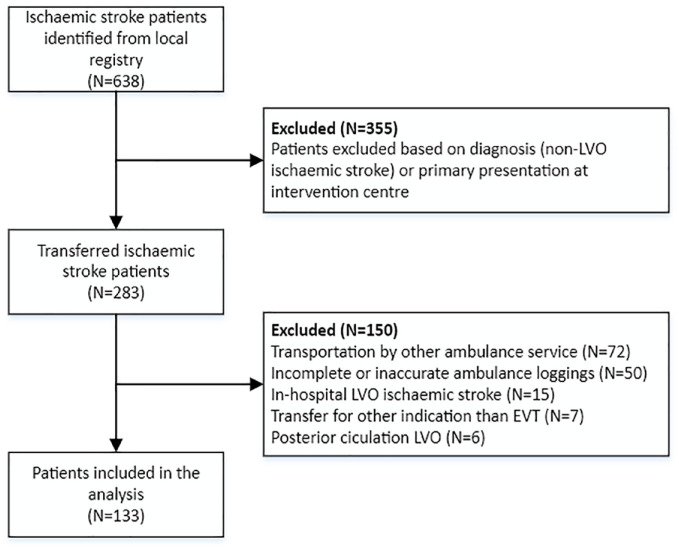
Flowchart of inclusion.

Between October 1, 2019, and November 30, 2020, 638 consecutive patients with acute ischaemic stroke were identified. After exclusion, 133 patients were identified for the analysis (Figure 1). Median (IQR) age was 74 (63–84) years and 61 (46%) patients were female, with a median baseline NIHSS of 14 (8–19) at the intervention centre. In the overall cohort, median (IQR) DIDO time was 66 (52–83) min (Table 1). In 125 patients with available imaging times, median (IQR) DIDO time was 66 (53–84), door-to-non-contrast enhanced CT-scan (NCCT) time was 16 (12–20) min, NCCT-to-CTA time was 9 (6–11) min, CTA-to-ambulance notification time was 24 (16–37) min and notification-to-departure time was 15 (10–21) min. Mean (±SD) CTA-to-ambulance notification time was the longest component of mean DIDO time with 29 (±20) min (38.7%) (Table 2).

**Table 1.** Cohort characteristics.

	Overall
*n*	133
*Clinical characteristics*	
Age (median (IQR))	74 (63–84)
Sex (%)	
Female	61 (46%)
Medical history (%)	
Atrial fibrillation	31 (23%)
Hypertension	78 (59%)
Hypercholesterolaemia	80 (60%)
Diabetes	30 (23%)
Ischaemic stroke or TIA	23 (17%)
Myocardial infarction	17 (13%)
Pre-stroke mRS (%)	
mRS 0–2	110 (83%)
mRS 3–5	23 (17%)
*Prehospital characteristics*	
Onset-to-door time (median (IQR))*	104 (48–357)
Last known well-to-door time (median (IQR))	428 (178–747)
Witnessed onset-to-door time (median (IQR))	54 (39–83)
Onset-to-door time within 6 h (%)	100 (75%)
Type of onset (%)	
Last known well	57 (43%)
Witnessed onset	76 (57%)
*Hospital characteristics and assessments*	
Primary stroke centre (%)	
Hospital 1	32 (24%)
Hospital 2	21 (16%)
Hospital 3	16 (12%)
Hospital 4	35 (26%)
Hospital 5	19 (14%)
Hospital 6	10 (8%)
Presentation during office hours (%)	60 (45%)
Blood pressure	
Systolic blood pressure, mmHg (mean (SD))	151 (25.5)
Diastolic blood pressure, mmHg (mean (SD))	87 (17.5)
Hypertension (%)	17 (14%)
Administration of IVT at PSC (%)	65 (49%)
NIHSS at intervention centre (median (IQR))	14 (8–19)
*Imaging characteristics*	
Acquisition of CTP at PSC (%)	29 (22%)
Occlusion side (%)	
Left	71 (53%)
Right	62 (47%)
Occlusion segment (%)	
ICA-T/M1	77 (58%)
M2	56 (42%)
*Logistical and procedural characteristics*	
Location of initial patient workup (%)	
CT-room	101 (76%)
ED-room	32 (24%)
Location of CT-room (%)	
ED based	54 (41%)
Elsewhere in hospital	79 (59%)
Transfer notification order (%)	
Ambulance first	56 (42%)
Intervention centre first	77 (58%)
Stroke alarm notification (%)	
ED notification	85 (64%)
Neurologist notification	48 (36%)
Workflow times, min	
Door-in-door-out time (median (IQR))	66 (52–83)
Door-in-door-out time within 1 h (%)	50 (38%)
Final treatment	
Endovascular thrombectomy (EVT) (%)	105 (79%)
Digital subtraction angiography (DSA) behind digital subtraction angiography without EVT (%)	5 (4%)
Groin-puncture without DSA or EVT (%)	1 (1%)
No DSA or EVT (%)	22 (16%)

IQR: interquartile range; TIA: transient ischaemic attack; mRS: modified Rankin Scale; SD: standard deviation; IVT: intravenous thrombolysis; PSC: primary stroke centre; NIHSS: National Institutes of Health Stroke Scale; CTP: computed tomography perfusion scan; NCCT: non-contrast-enhanced computed tomography scan; CTA: computed tomography angiography scan.

Data are reported as *n* (%), mean (SD) or median (IQR).

* Onset time is defined as either time of symptom onset if witnessed, or last known well if unwitnessed. Missing data: Systolic blood pressure: 10, diastolic blood pressure: 10, hypertension: 10, NCCT-acquisition time: 5, CTA-acquisition time: 7, door-to-NCCT time: 5, NCCT-to-CTA time: 8, CTA-to-ambulance notification time: 7. All time intervals were known in 125 patients.

**Table 2.** Composition of door-in-door-out time in patients for whom data was available to reconstruct all time intervals (*N* = 125).

Workflow interval	Median time (IQR), min	Mean time (±SD), min	Percentage of total
Door-in-door-out time	66 (53–84)	75 (±32)	100
Door-to-NCCT time	16 (12–20)	21 (±22)	28.0
NCCT-to-CTA time	9 (6–11)	10 (±8)	13.3
CTA-to-ambulance notification time	24 (16–37)	29 (±20)	38.7
Notification-to-departure time	15 (10–21)	15 (±8)	20.0

SD: standard deviation; IQR: interquartile range; NCCT: non-contrast-enhanced computed tomography scan; CTA: computed tomography angiography scan.

Data are reported as mean (SD) or median (IQR).

In univariable linear regression analysis, the following variables showed a significant association with DIDO time (*p*-value <0.20): age (+4 min per 10 years, 95% CI: 0.01–8); type of onset (+13 min for last known well, 95% CI: 3–24); onset-to-door time longer than 6 h (+22 min, 95% CI: 10–33); treatment with IVT (−16 min if treated, 95% CI: −26 to −6); NIHSS at intervention centre (−0.8 min per point, 95% CI: −2 to −0.02); occlusion segment (+15 min for M2-occlusion compared to ICA-T/M1 occlusion, 95% CI: 4–26); and side of occlusion (+11 min for right-sided occlusion, 95% CI: 0.1–21). Sex, pre-stroke mRS, presence of hypertension, time of presentation, transferring PSC, acquisition of CTP, location of initial patient workup, location of CT-room, transfer notification order and stroke alarm notification showed no significant relation with DIDO time at the prespecified significance level of *p* < 0.20 (Table 3).

**Table 3.** Fixed effects of the linear mixed regression model for the association between determinants and door-in-door out time (min).

Door-in-door-out time, min	Univariable regression coefficient (95% CI)	Multivariable regression coefficient (95% CI)
Age		
Per 10 years	3.9 (0.01 to 7.9)	5.6 (2.1 to 9.1)
Sex		
Male	*Reference*	–
Female	3.3 (−7.5 to 14.1)	–
Pre-stroke mRS		
mRS 0–2	*Reference*	–
mRS 3–5	3.3 (−10.9 to 17.6)	–
Hypertension at PSC		
No	*Reference*	–
Yes	4.8 (−11.1 to 20.7)	–
Time of presentation		
During office hours	*Reference*	–
Outside office hours	2.5 (−8.4 to 13.3)	–
Type of onset		
Witnessed	*Reference*	*Reference*
Last known well	13.2 (2.5 to 23.8)	1.1 (−10.9 to 13.2)
Onset-to-door time		
Within 6 h	*Reference*	*Reference*
Outside 6 h	21.5 (9.6 to 33.4)	19.8 (4.9 to 34.6)
Administration of IVT		
No	*Reference*	*Reference*
Yes	−16.0 (−26.4 to −5.6)	−7.7 (−18.6 to 3.2)
Acquisition of CTP		
No	*Reference*	–
Yes	5.2 (−7.8 to 18.2)	–
NIHSS at intervention centre		
Per point	−0.8 (−1.5 to −0.02)	−0.4 (−1.2 to 0.4)
Occlusion side		
Left	*Reference*	*Reference*
Right	10.8 (0.1 to 21.4)	11.9 (2.5 to 21.3)
Occlusion segment		
ICA-T/M1	*Reference*	*Reference*
M2	15.0 (4.4 to 25.6)	14.9 (4.1 to 25.6)
Location of initial patient workup		
CT-room	*Reference*	–
ED-room	2.1 (−10.5 to 14.7)	–
Location of CT-room		
Emergency department	*Reference*	–
Elsewhere in hospital	−3.6 (−14.6 to 7.3)	–
Transfer notification order		
Ambulance first	*Reference*	–
Intervention centre first	−1.6 (−12.5 to 9.3)	–
Stroke alarm notification		
Emergency department	*Reference*	–
Neurologist	2.9 (−8.3 to 14.1)	–

CI: confidence interval; mRS: modified Rankin Scale; PSC: primary stroke centre; IVT: intravenous thrombolysis; CTP: computed tomography perfusion scan; NIHSS: National Institutes of Health Stroke Scale.

Data reported are coefficients with 95% confidence interval.

In the multivariable linear mixed regression analysis, age (+6 min per 10 years, 95% CI: 2–9), onset-to-door time longer than 6 h (+20 min, 95% CI: 5–35), occlusion segment (+15 min for M2-occlusion compared to ICA-T/M1 occlusion, 95% CI: 4–26) and right-sided occlusion (+12 min, 95% CI: 2–21) were independently associated with DIDO time (Table 3). There was no variation between primary stroke centres in DIDO time (variance of the random intercepts was 0).

## Discussion

DIDO times are an important cause of treatment delay in patients eligible for EVT. Identification of bottlenecks and determinants may help minimize DIDO times and improve functional outcome in patients with acute ischaemic stroke caused by LVO. We found that CTA-to-ambulance notification time was the largest component of DIDO time. Higher age, onset-to-door time longer than 6 h, right-sided occlusion and M2-occlusion were independent predictors of longer DIDO time.

It is not surprising that the CTA-to-ambulance notification time is the longest time interval, as this window encompasses many instances of decision-making, consultations and transfers of information (i.e. interpreting imaging, explaining the findings to patient and family, obtaining informed consent for treatment). Higher age and onset-to-door time longer than 6 h may lead to a prolonged DIDO time because the sense of urgency is lower when compared to younger patients and patients who arrive within the conventional treatment window for EVT in acute ischaemic stroke. A possible explanation for right-sided occlusion as a determinant of longer DIDO time may be that aphasia, which is a telltale clinical sign for LVO often incorporated in prehospital stroke scales, is attributed to lesions in the left hemisphere in the majority of patients.^13–15^ Given their anatomical location, M2-occlusions are generally more difficult to recognize on imaging than ICA-T/M1-occlusions, which explains why a more distal occlusion may lead to diagnostic delay and thus a longer DIDO time.^16^

In recent years, many studies regarding in-hospital workflow times for patients with acute ischaemic stroke have been published, mainly focussing on door-to-needle times, door-to-groin times and door-to-reperfusion times. In contrast, few studies have been published on DIDO times of acute ischaemic stroke patients, nor are DIDO times routinely collected as a quality indicator for acute stroke care. A previous cohort study conducted in Australia, including 67 patients found a median DIDO time of 106 min. CT-to-ambulance notification was the largest component of DIDO time, while PSC site, presentation during working hours and re-recruitment of initial ambulance crew for transportation of patient to the intervention centre were associated with a shorter DIDO time.^17^ Another cohort study conducted in Australia, did not identify statistically significant determinants for DIDO time in 55 patients with a median DIDO time of 120.5 min.^18^ Finally, a more recent cohort study conducted in the United States found a median DIDO time of 148.5 min in 191 patients, where CTA acquisition at PSC, walk-in arrival mode, administration of IVT, intubation at PSC and ambulance request by PSC were independently associated with DIDO time.^19^ Generally, the median DIDO times in these studies were long and study populations small. We reproduced the finding that CT-to-ambulance notification is the largest component of DIDO time, but we did not find that presentation during office hours, referring PSC site or administration of IVT were independently associated with DIDO time. It is probable that regional practices and healthcare organization play an important role in the length, composition and determinants of DIDO time. The effects of these influential regional factors on DIDO time are probably magnified in studies with longer DIDO times. This leads to a limited generalizability of these findings to other regions. Other regions with long DIDO times likely face their own region-specific factors that lead to a less efficient work process, while regions with shorter DIDO times have likely already addressed these mutable causes. This point is illustrated by the findings of one of the previous studies that walk-in arrivals and CTA acquisition at PSC were associated with longer DIDO time.^19^ In our study region, walk-in arrivals are very uncommon and CTA is part of the routine workup for every stroke patient at a PSC. Because the influence of these factors has already been negated in our study region, we were unable to investigate the relationship between these factors and DIDO time. This may imply a more advanced stage of process improvement within our study region. We also did not find significant differences in DIDO times between centres, which may be an indication of a more regionally harmonized working process when compared to other regions. The relatively short DIDO time, regionally harmonized working process and the apparent absence of influential region-specific practices in our region, may make our results more generalizable to other regions and hospitals with an equivalent or less advanced stage of process improvement.

Another potential target for reducing DIDO time is the ambulance notification-to-departure time. Implementation of criteria to have the initial ambulance crew wait for a potential transfer, such as a certain stroke severity threshold, may have a substantial effect on DIDO time. However, more insight into the trade-off between expedited transfers and wasted ambulance crew-hours is required before such a recommendation could be made.

For clinicians aiming to improve DIDO times at their own centres, our findings may form a basis for process improvement, but it remains important to critically review the local workflow process and to identify potential bottlenecks causing long DIDO times. A benchmark DIDO time of 1 h or less should be attainable, especially given the large proportion of patients in which DIDO times under 60 min were already achieved (Table 1). There is no doubt that efforts to decrease DIDO time can lead to shorter onset-to-groyne times and better functional outcomes in patients requiring interhospital transfers for EVT.

This study has a few limitations. Due to the retrospective design, some potential determinants were not readily available, such as the NIHSS at PSC. Instead, the NIHSS at the intervention centre, which is routinely collected for all stroke patients undergoing EVT, served as a proxy for initial stroke severity. Another limitation is that the time of departure was registered manually. Some fluctuations in DIDO times may be caused by variation in the reporting of departure times. However, as the arrival at intervention centre was automatically registered, we could reconstruct driving times as the interval between departure of PSC and arrival at intervention centre. The observed variations in driving times were minimal overall, with the exception of a few outliers (data not shown due to centre pseudonymisation). Finally, we only have data from a single ambulance region, which prohibits us from further exploring the influence of ambulance service-specific parameters on DIDO time.

Given these findings, it is advisable to focus on faster recognition of M2-occlusions and raise the level of awareness for patients with a longer onset-to-door time, especially as extended window EVT is quickly gaining ground.^20,21^ Further research to identify determinants of longer DIDO times is warranted. Preferably, these studies should gather data prospectively, in order to more accurately establish the various time intervals and determinants.

## Conclusion

The CTA-to-ambulance notification time is the longest contributing interval, making up almost 40% of DIDO time. Higher age, onset-to-door time longer than 6 h, M2-segment occlusion and right-sided occlusions are independently associated with a longer DIDO time. Interventions that aim to decrease DIDO time should take these findings into account.

## Supplemental Material

sj-doc-1-eso-10.1177_23969873231177768 – Supplemental material for Determinants of door-in-door-out time in patients with ischaemic stroke transferred for endovascular thrombectomy Click here for additional data file. Supplemental material, sj-doc-1-eso-10.1177_23969873231177768 for Determinants of door-in-door-out time in patients with ischaemic stroke transferred for endovascular thrombectomy by Ruben M van de Wijdeven, Martijne HC Duvekot, Patrick J van der Geest, Walid Moudrous, Kirsten RIS Dorresteijn, Annemarie D Wijnhoud, Laus JMM Mulder, Kees CL Alblas, Nabil Asahaad, Henk Kerkhoff, Diederik WJ Dippel and Bob Roozenbeek in European Stroke Journal
